# Anti-Influenza Activity of an Ethyl Acetate Fraction of a *Rhus verniciflua* Ethanol Extract by Neuraminidase Inhibition

**DOI:** 10.1155/2020/8824934

**Published:** 2020-10-29

**Authors:** Young Soo Kim, Wei Li, Ji Hye Kim, Hwan-Suck Chung, Jang-Gi Choi

**Affiliations:** Korea Institute of Oriental Medicine (KIOM), Korean Medicine (KM) Application Center, 70 Cheomdan-ro, Dong-gu, Daegu 41062, Republic of Korea

## Abstract

Antigenic mismatch can cause influenza vaccines to be ineffective, and influenza viruses resistant to antiviral drugs are rising. Thus, development of antiviral agents against these viruses is an immediate need. *Rhus verniciflua* (RVS) has long been used in herbal medicine and as a nutritional supplement. The effect of RVS and its components on influenza virus has not, however, been reported. We found that RVS treatment significantly reduced viral replication when evaluated with green fluorescent protein- (GFP-) tagged virus (influenza A virus, A/PR/8/34-GFP) in Madin–Darby canine kidney (MDCK) cells. RVS showed significant inhibition of neuraminidase from A/PR/8/34. Subsequently, three fractions were prepared from an ethanolic crude extract of RVS. *In vitro* assays indicated that an ethyl acetate fraction (RVSE) was more potent than H_2_O and CHCl_3_ fractions. RVSE significantly suppressed influenza virus infection in MDCK cells via neuraminidase inhibition. Additionally, RVSE treatment inhibited expression of several virus proteins and decreased mortality of mice exposed to influenza A/PR/8/34 by 50% and reduced weight loss by 11.5%. Active components in RVSE were isolated, and 5-deoxyluteolin (5) and sulfuretin (7) demonstrate the highest neuraminidase inhibitory activity against influenza A virus. RVS, RVSE, and their constituents may be useful for the development of anti-influenza agents.

## 1. Introduction

Seasonal influenza virus (IV) infects 5%–15% of the global human population each year and kills about 500,000 people [1]. Coronavirus 2 (SARS-CoV-2) is currently a serious global public health crisis. Coinfection of SARS-CoV-2 and influenza virus is common during periods of increased novel coronavirus disease (COVID-19) [2–4]. Patients who are coinfected with SARS-CoV-2 and influenza viruses are at high risk for poor outcomes [5]. To date, two types of anti-influenza drug, matrix protein 2 (M2) ion channel blockers [6] and neuraminidase (NA) inhibitors [7, 8], have been approved. Another antivirus drug, an RNA polymerase inhibitor, is now regionally approved [9, 10]. NA inhibitors, such as oseltamivir and zanamivir, are frequently prescribed, whereas M2 ion channel blockers, such as amantadine, are rarely used because of the emergence of resistant influenza strains [11–13].

NA is a glycoprotein present on the surface of influenza viruses and is required for release of progeny virions from infected cells. NA acts by cleaving sialic acid groups on cell surfaces that bind to viral hemagglutinin. Thus, NA inhibitors prevent progeny virions from budding from infected cells. The active site of NA is highly conserved in both influenza A and B [14–16]. However, H274Y and E119G/D/A mutations in the NA gene decrease susceptibility to NA inhibitors oseltamivir and zanamivir, respectively. Such resistance leads to the current demand for the development of new NA inhibitors [17, 18].

Phytochemicals from medicinal plants provide valuable building blocks for new drug development [19, 20]. *Rhus verniciflua* Stokes (RVS), which produces various bioactive constituents, has been used as a traditional herbal medicinal plant for various diseases, such as gastroenteritis, diabetes, arthritis, hypertension, stroke, and cancer. Aromatic compounds from RVS significantly block PD-1/PD-L1 and CTLA-4/CD80 interactions [21]. Antiviral efficacy of RVS has been investigated for human immunodeficiency virus type 1 and fish pathogenic viruses, but not for influenza [22, 23].

Thus, we examined the effects of an RVS ethyl acetate fraction (RVSE) on inhibiting the replication of influenza virus *in vitro* and *in vivo*. We initially assessed the potential of RVSE to inhibit influenza virus replication and underlying mechanisms of action *in vitro*, focusing on the inhibition of NA activity. Subsequently, we investigated RVSE for protection of mice from a lethal challenge with influenza virus. RVSE significantly averted influenza virus infection in Madin–Darby canine kidney (MDCK) cells via inhibition of NA. RVSE treatment also decreased mortality and prevented weight loss in mice exposed to influenza A/PR/8/34 virus. We also isolated and identified 10 major components in the RVSE and found that 5-deoxyluteolin (5) and sulfuretin (7) demonstrated the highest NA inhibitory activity. RVS, RVSE, and their components were effective in inhibiting the NA activity of both influenza virus A and B, suggesting that RVSE and its components may be good candidates and building block for novel anti-influenza drugs.

## 2. Materials and Methods

### 2.1. Plant Material

Dried bark of RVS was kindly provided from Bomyeong Herbal Market, Seoul, in 2018. Its identity as *R*. *verniciflua* was confirmed by one of the authors (Dr. Wei Li). A voucher specimen (IC-180018) was deposited at the Herbarium of Korean Medicine-Application Center, Korea Institute of Oriental Medicine, Republic of Korea.

### 2.2. Preparation of RVS and RVSE

Dried bark of RVS (8.0 kg) was exhaustively extracted under reflux with 70% ethanol three times, each time with 50 L solvent. The total extract (330.0 g) was suspended in deionized water and partitioned with CHCl_3_ (80.0 g). The water fraction was then partitioned sequentially with ethyl acetate (EA) (125.0 g).

### 2.3. Cells and Viruses

MDCK and A549 human lung epithelial cells were obtained from the American Type Culture Collection and maintained in Dulbecco's modified eagle medium (DMEM) (Lonza, Walkersville, MD, USA) containing 10% fetal bovine serum (FBS; Biotechnics Research, Lake Forest, CA, USA) and 1% each of penicillin and streptomycin (Cellgro, Manassas, VA, USA) at 37°C in a 5% CO_2_ incubator. Influenza virus strains were grown and titrated, as previously described [24]. In this work, we used influenza A/Puerto Rico/8/34 (A/PR/8/34) and green fluorescent protein- (GFP-) tagged A/PR/8/34 (A/PR/8/34-GFP) viruses, also used in previous studies [24–26]. Briefly, A/PR/8/34-GFP was constructed by fusing the GFP gene to the C-terminal end of nonstructural protein 1 (NS-1) open reading frame, containing the silent mutation at the splice acceptor, without the stop codon, and followed by the autoproteolytic site and nuclear export protein. Other influenza A strains (H1N1, A/Korea/33/2005; H3N2, A/Korea/32/2005) and influenza B (B/Korea/72/2006) viruses were purchased from the Korea Bank for Pathogenic Viruses.

### 2.4. Reagents

Oseltamivir carboxylate was purchased from AOBIOUS Inc. (Gloucester, MA, USA). Antibodies targeting influenza proteins, PA, NA, NP, PB1, PB2, M1, and NS-1, were procured from GeneTex (San Antonio, TX, USA). Anti-*β*-actin was purchased from Cell Signaling Technology (Cell Signaling Technology, Boston, MA, USA).

### 2.5. MTS Assay

Cell viability was determined using the CellTiter 96® AQueous One Solution Cell Proliferation Assay (Promega, Madison, WI, USA), following the manufacturer's instructions. MDCK cells (1 × 10^4^ cells/well) were seeded into 96-well plates, and RVS and RVSE were added to wells at concentrations of 0–400 *μ*g/mL. After 48 h, MTS solutions were added to each well, and the cells were incubated for additional 2 h. Subsequently, absorbance at 490 nm was recorded using a GloMax® Explorer Multimode Microplate Reader (Promega, Madison, WI, USA). The values of MTS assay were represented by the mean ± SEM of four independent experiments.

### 2.6. Antiviral Assay

The inhibition of viral replication was assayed, as previously described [27]. Briefly, MDCK cells were cultured in 24-well plates (1 × 10^5^ cells/well) for 16 h. Differing RVSE concentrations (100 or 200 *μ*g/mL) were added to H1N1 (multiplicity of infection (MOI) = 1) and A/PR/8/34-GFP (MOI = 1), and the mixtures were incubated at 37°C for 1 h. MDCK cells were infected with these mixtures at 37°C for 2 h. Afterwards, the virus was removed, cells were washed three times with phosphate-buffered saline (PBS), and the medium was replaced by complete DMEM. The cells were incubated for 48 h at 37°C with 5% CO_2_. Influenza virus GFP expression was measured under a fluorescence microscope (Olympus, Tokyo, Japan) following 24 h of viral infection. In addition, antiviral activities of RVSE components were evaluated at a concentration of 100 *μ*M by the same method described earlier. Antiviral assays are presented as mean ± SEM of four (RVSE) and three (RVSE components) independent experiments.

### 2.7. Analysis of GFP Expression Using Flow Cytometry

MDCK or A549 cells were cultured in 24-well plates (1 × 10^5^ cells/well) for 18 h. A/PR/8/34 (MOI = 1) was mixed with different concentrations of RVS and RVSE (0, 12.5, 25, 50, and 200 *μ*g/mL), and the mixtures were incubated at 37°C for 1 h. MDCK and A549 cells were infected with these mixtures at 37°C for 2 h. Subsequently, the virus was removed, and the cells were washed three times with PBS, and the medium was replaced by complete DMEM. Cells were incubated for 24 h at 37°C with 5% CO_2_. Reduction of viral infection was determined by measuring GFP expression using flow cytometry. MDCK or A549 cells were harvested and resuspended in 1 mL of PBS containing 2% FBS and fixed in suspension with 4% paraformaldehyde. The cells were washed three times with PBS and stored at 4°C until analysis with a CytoFLEX flow cell counter (Beckman). We analyzed data using FlowJo software.

### 2.8. NA Inhibition (NI) Assay

The NI assay was performed using an NA-Fluor™ Influenza Neuraminidase Assay Kit (Applied Biosystems, Foster City, CA, USA) following the manufacturer's instructions with slight modifications [28, 29]. RVSE was added to assay buffer in 96-well plates at concentrations of 0–400 *μ*g/mL for A/PR/8/34, H3N2, H1N1, and influenza type B viruses. A/PR/8/34, H1N1, H3N2, or influenza type B in assay buffer was added to RVS- and RVSE-containing wells and incubated at 37°C. Oseltamivir was considered a positive control in the assay. After 30 min, NA-Fluor Substrate was added to each well and incubated for additional 2 h, followed by recording fluorescence (excitation, 365 nm; emission, 415–445 nm) with a fluorescence spectrophotometer (Promega, Madison, WI, USA). Samples treated with only RVSE or its components were used as negative controls. Further, NA activities for 10 *μ*M concentrations of RVSE components were evaluated by the same method described earlier. NA activity after incubation with oseltamivir carboxylate was measured in a range of 0–10,000 nM as a positive control. NI assay results are presented as mean ± SEM of three independent experiments.

### 2.9. Isolation Procedures

The nuclear magnetic resonance (NMR) spectra were recorded using a Bruker Avance III 600 NMR spectrometer (^1^H, 600 MHz; ^13^C, 150 MHz) (Bruker BioSpin GmbH, Karlsruhe, Germany), with tetramethylsilane as an internal standard. Heteronuclear multiple quantum correlation, heteronuclear multiple bond correlation, rotating frame nuclear overhauser effect spectroscopy, and ^1^H–^1^H correlation spectroscopy spectra were recorded using a pulsed-field gradient. Preparative HPLC used a Gilson 321 pump, a 151 UV/VIS detector (Gilson SAS, Villiers-le-Bel, France), and an RS Tech HECTOR-M C18 column (5 *μ*m particle size, 250 × 21.2 mm) (RS Tech Corp, Chungju, South Korea). Column chromatography was performed using silica gel (Kieselgel 60, 70–230, and 230–400 mesh; Merck, Darmstadt, Germany) and YMC C18 resin. Thin-layer chromatography was performed using precoated silica gel 60 F_254_ and RP-18 F_254S_ plates (both 0.25 mm thickness; Merck, Darmstadt, Germany). Spots were detected under UV light and using 10% H_2_SO_4_.

### 2.10. Isolation of Chemicals

The EA fraction was subjected to silica gel column chromatography with a gradient of CHCl_3_–methanol (MeOH)–H_2_O (30 : 1 : 0 to 1.5 : 1 : 0.15, MeOH) to give 12 fractions (fractions A–L). Fraction C was separated on a silica gel column (2.5 × 80 cm) with a gradient of CHCl_3_–acetone (5%–50%) to give six subfractions (C1–C6). Fraction C3 was isolated by preparative HPLC (MeOH–H_2_O: 35%) to give component 6 (880.0 mg). Fraction G was separated on a silica gel column (3.0 × 80 cm) with a gradient of CHCl_3_–acetone (5%–55%) to give four subfractions (G1–G4). Fraction G2 was isolated by preparative HPLC (acetone–H_2_O: 10%–30%) to give components 1 (2.02 g) and 9 (45.0 mg). Fraction G4 was isolated by preparative HPLC (acetone–H_2_O: 5%–30%) to give components 10 (11.0 mg) and 4 (50.0 mg). Fraction I was isolated with a gradient of MeOH–H_2_O (15%–25%) by medium pressure liquid chromatography (MPLC) using a YMC C18 column to give components 2 (255.0 mg) and 3 (15.0 mg). Fraction J was isolated with a gradient of MeOH–H_2_O (20%–25%) by MPLC using a YMC C18 column to give component 5 (680.0 mg). Fraction L was separated on a silica gel column (1.5 × 80 cm) with a gradient of CHCl_3_–acetone (3%–45%) to give three subfractions (L1–L3). Fraction L1 was isolated by preparative HPLC (acetone–H_2_O: 10F50%) to give component 7 (440.0 mg). Fraction L3 was isolated by preparative HPLC (acetone–H_2_O: 10%–50%) to give component 8 (5.5 mg).

### 2.11. HPLC Analysis

Two RVSE components 5 and 7 were analyzed using an Alliance e2695 (Waters Corp., Milford, MA, USA) with injection of 10 *μ*L of RVS (5 mg/mL), RVSE (1 mg/mL), and standard samples into a Gemini C18 column (5 *μ*m, 250 × 4.6 mm; Phenomenex Inc., Torrance, CA, USA) at an oven temperature of 40°C. The mobile phase was applied at a flow rate of 1 mL/min with a gradient of acetonitrile (A) and distilled water (B) containing 1% acetic acid as follows: 5% A (0–3 min), 5%–100% A (3–60 min), 100% A (60–62 min), 100%–5% A (62–63 min), and then 5% A (63–68 min). The samples were monitored under UV light at 254 nm.

### 2.12. Docking Simulation and Interaction Analysis

Two RVSE components 5 and 7 were docked onto the predefined binding pocket of the H1N1 NA crystal structure (PDB code: 3TI6) retrieved from the Protein Data Bank (www.rcsb.org) using SwissDock [30]. After docking simulation, the lowest energy scoring binding mode for each component was selected. The hydrogen bonding and hydrophobic interactions between H1N1 NA and each component were investigated with LigPlot+ v1.4.5 [31]. Amino acid residues involved in interactions were indicated with green (H-bonds) and red (hydrophobic interactions).

### 2.13. Immunofluorescence Staining

For the immunofluorescence analysis, we used a slightly modified version of a previously used immunofluorescence analysis method [24]. Briefly, MDCK cells were cultured in 4-well tissue culture slides (1 × 10^5^ cells/well) for 18 h. Subsequently, A/PR/8/34-GFP (MOI = 5) were mixed with different concentrations of RVSE (25 and 100 *μ*g/mL), and the mixtures were incubated at 37°C for 1 h. MDCK cells were infected with these mixtures at 37°C for 2 h. Thereafter, the virus was removed, and the cells were washed three times with PBS and were cultured in a CO_2_ incubator at 37°C for 24 h. Cells were then washed three times with cold PBS and fixed with 4% paraformaldehyde in PBS and 1% Triton X-100 for 10 min each at room temperature. After blocking, the fixed cells were incubated overnight at 4°C with M2-specific antibodies, washed three times (5 min per wash) with TBS, and incubated with Alexa Fluor 568 goat anti-rabbit IgG antibody (1 : 1,000; Life Technologies, Eugene, OR, USA) and washed three times (5 min per wash) with TBS. Next, the cells were incubated with DAPI for 10 min and measured using fluorescence microscopy.

### 2.14. Western Blot Analysis

MDCK cells were cultured in 6-well plates (1 × 10^6^ cells/well) for 18 h. H1N1 was mixed with different concentrations of RVSE (12.5, 25, 50, and 100 *μ*g/mL), and the mixtures were incubated at 37°C for 1 h. MDCK cells were infected with these mixtures at 37°C for 2 h. Afterwards, the virus was removed, cells were washed three times with PBS, and the medium was replaced by complete DMEM. After 24 h, cells were harvested and subjected to western blotting using whole cell extracts [24]. The PVDF membrane was then blocked with 5% BSA in TBS-T buffer for 1 h and incubated overnight at 4°C with primary anti-PA, anti-NA, anti-NP, anti-PB1, anti-PB2, anti-M1, and anti-NS-1 and anti-*β*-actin antibodies (1 : 1,000 dilution). Primary antibodies were washed three times (5 min per wash) with TBS-T buffer and incubated with HRP-conjugated secondary antibodies (1 : 5,000 dilution) at room temperature for 1 h. Relative intensities of protein bands were measured using ImageJ program [24]. The experiment was repeated independently three times, and similar results were obtained in each replicate.

### 2.15. Viral Yield Reduction Assay

To investigate the inhibition of viral replication by RVSE, we used a slightly modified version of a previously described viral yield reduction assay measuring the virus-induced red blood cell (RBC) hemolysis [32]. Briefly, MDCK cells were cultured in 24-well plates (1 × 10^5^ cells/well) for 24 h. Subsequently, H1N1 (MOI = 1) was mixed with different concentrations of RVSE (0, 12.5, 25, 50, and 100 *μ*g/mL), and the mixtures were incubated at 37°C for 1 h. MDCK cells were infected with these mixtures at 37°C for 2 h. The culture medium was used as a negative control. Briefly, 50 *μ*L PBS was added to each well of a U-bottomed 96-well plate. Each cell culture supernatant was serially diluted twofold in the previously loaded PBS. Finally, 100 *μ*L 1% chicken RBCs were added to each well. Assays were evaluated over the course of a 1 h incubation at room temperature. RBCs in negative wells sedimented and exhibited agglutination. Positive wells had an opaque appearance or displayed hemolysis with no sedimentation. Titers are presented in hemagglutination units/50 *μ*L (HAU/50 *μ*L) in comparison with those of the control treatment [32].

### 2.16. Animal Studies

This study was carried out in accordance with the guidelines of the Institutional Animal Care and Use Committee (IACUC) of the Laboratory Animal Center of Daegu-Gyeongbuk Medical Innovation Foundation (DGMIF). Animal studies were approved by the IACUC of the Laboratory Animal Center of DGMIF under approval number DGMIF-17031401-01. Female 5-week-old BALB/c mice from Orient Bio Inc. (Seongnam, South Korea) were acclimated for at least 1 week under standard housing conditions at DGMIF. Mice were provided standard rodent chow and water *ad libitum*. For oral inoculation of RVSE and influenza A virus challenge, mice were separated into three experimental sets each with three groups of 10 mice (PBS, RVSE (10 mg/kg) with virus infection, and PBS with virus infection). Mice in the latter two groups were orally administered 10 mg/kg RVSE in a volume of 200 *μ*L once daily for 10 days post infection (dpi). Mice were infected intranasally with five times the 50% of the lethal dose for mice (LD_50_) of A/PR/8/34 in 20 *μ*L of PBS. Body weight and survival were monitored for 10 dpi at fixed time points [32].

### 2.17. Statistical Analysis

Data are expressed as mean ± SEM. Differences in mean values between the treatment and control groups were statistically significant using one-way ANOVA with Tukey's post hoc test for multiple comparisons. Analyses were performed using GraphPad PRISM software® Version 5.02 (GraphPad, La Jolla, CA, USA); *p* < 0.05 denoted statistical significance.

## 3. Results

### 3.1. Inhibition of NA Activity by RVS and Its Fractions

**Figure 1:**
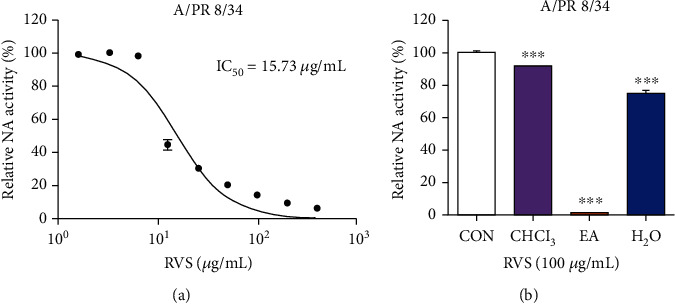
Measurement of the antiviral activity of RVS and fraction (chloroform (CHCl_3_) and water (H_2_O)) using neuraminidase (NA) inhibition assay. Influenza A viruses, including A/PR/8/34, were added to indicated (a) concentrations of RVS and (b) its fractions. Fluorescence was measured using fluorescence spectrophotometry (excitation, 365 nm; emission, 415–445 nm). Bar graph (mean ± SEM) statistics were determined with data from three experiments using one-way ANOVA with Tukey's post hoc test, ^∗∗∗^*p* < 0.001; ^∗∗^*p* < 0.01. n.s.: not significant, compared with the RVSE untreated samples.

**Figure 2:**
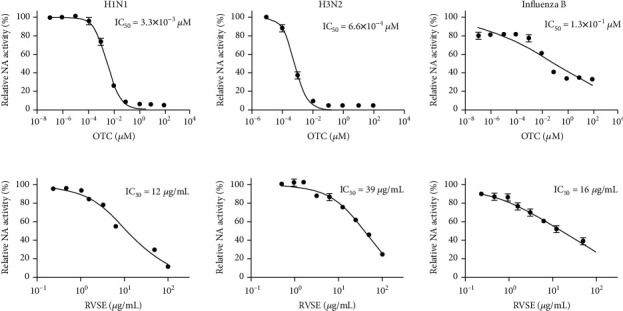
Measurement of the antiviral activity of RVSE using NA inhibition assay. Influenza A viruses including H1N1, H3N2, and influenza type B were added to indicated concentrations of RVSE or oseltamivir carboxylate (OTC). Fluorescence was measured using fluorescence spectrophotometry (excitation, 365 nm; emission, 415–445 nm).

NI is recognized as a quality anti-influenza drug target that prevents progeny virions from being released from infected cells. We investigated the ability of RVS and its fractions for inhibition of NA activity. NI assay showed that RVS effectively inhibited NA activity of A/PR/8/34 at concentrations above 12.5 *μ*g/mL (Figure 1(a)). Additionally, we confirmed that the EA fraction of RVS inhibits NA activity of H1N1 (A/PR/8/34) by 98.3%. This inhibition is superior to other fractions (chloroform, 8.0%; water, 24.5%) (Figure 1(b)). We also assessed the inhibition of NA activity by RVSE and a positive control oseltamivir carboxylate using various influenza viruses: H1N1 (A/PR/8/34 and A/Korea/33/2005), H3N2 (A/Korea/32/2005), and influenza type B (B/Korea/72/2006). RVSE caused a significant decrease in NA activity in a dose-dependent manner (Figure 2). In particular, NA from influenza type B NA was 0.8- and 2.4-fold more susceptible to RVSE than NA from H1N1 and H3N2. Oseltamivir carboxylate was less effective toward NA from influenza type B. RVSE will have anti-influenza virus efficacy though inhibiting the release of progeny virions from infected cells.

### 3.2. Cell Viability of RVS-Treated MDCK Cells

**Figure 3:**
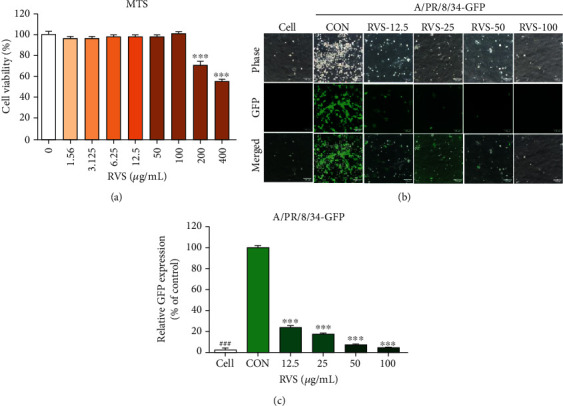
Determination of cytotoxicity and antiviral activity of *R*. *verniciflua* Stokes (RVS) ethanol extract in MDCK cells. Viability of MDCK cells was assessed using MTS assay after treatment with indicated concentrations of RVS (0–400 *μ*g/mL) for 48 h (a). Antiviral activities of RVS on influenza A/PR/8/34-GFP virus in MDCK cells. MDCK cells were treated with RVS (12.5, 25, 50, and 100 *μ*g/mL) before influenza A virus (A/PR/8/34-GFP) infection, and cells were incubated with medium alone (CON) or 12.5, 25, 50, and 100 *μ*g/mL of RVS before infection with A/PR/8/34-GFP (multiplicity of infection = 1) (b). GFP expression and reduction in viral replication using flow cytometry were assessed 24 h after viral infection in GHE-treated MDCK cells (c). Bar graph (mean ± SEM) statistics were determined with data from three experiments using one-way ANOVA with Tukey's post hoc test, ^∗∗∗^*p* < 0.001; ^∗∗^*p* < 0.01. n.s.: not significant, compared with the (RVSE untreated) samples.

Cytotoxicity of RVS was investigated by incubating MDCK cells with various concentrations (0–400 *μ*g/mL) for 48 h. MDCK cells did not show cytotoxicity. RVS concentration reached 100 *μ*g/mL (Figure 3(a)). The following experiments were conducted at an RVS concentration below 100 *μ*g/mL.

### 3.3. RVS Inhibited the Infection of Influenza Virus in MDCK Cells

MDCK cells treated with RVS concentrations of 0, 12.5, 25, 50, or 100 *μ*g/mL were infected with A/PR/8/34-GFP (Figure 3(b)). RVS-treated MDCK cells showed significantly reduced GFP expression levels compared with untreated cells 24 h after infection (Figure 3(b)). Additionally, flow cytometry analysis using fluorescence detection indicated that RVS effectively inhibits viral replication in MDCK cells (Figure 3(c)). RVS-treated cells showed a significantly reduced viral load following infection with influenza virus compared with untreated cells.

### 3.4. RVSE Inhibited Infection of MDCK Cells by Influenza Virus

**Figure 4:**
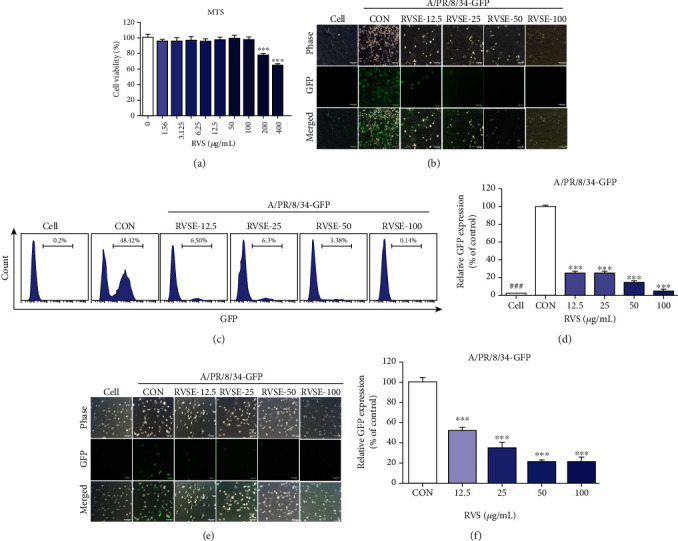
Determination of antiviral activity of RVS ethyl acetate fraction (RVSE) in MDCK and A549 cells. Viability of MDCK cells was assessed using MTS assay after treatment with indicated concentrations of RVSE (0–400 *μ*g/mL) for 48 h (a). Antiviral activities of RVSE on influenza A/PR/8/34-GFP virus in MDCK cells. MDCK cells were treated with RVSE (12.5, 25, 50, and 100 *μ*g/mL) before influenza A virus (A/PR/8/34-GFP) infection, and cells were incubated with medium alone (CON) or with 12.5, 25, 50, and 100 *μ*g/mL of RVSE before A/PR/8/34-GFP (multiplicity of infection = 1) (b). GFP expression levels and reduction in viral replication using flow cytometry were assessed 24 h after viral infection in GHE-treated MDCK cells (c, d). A549 cells were treated with RVSE (12.5, 25, 50, and 100 *μ*g/mL) prior to influenza A virus (A/PR/8/34-GFP) infection, and cells were incubated with medium alone (CON) or 12.5, 25, 50, and 100 *μ*g/mL of RVSE prior to infection with A/PR/8/34-GFP (multiplicity of infection = 1) (b). GFP expression levels and reduction in viral replication using flow cytometry were assessed at 24 h after viral infection in GHE-treated A549 cells (c, d). Bar graph (mean ± SEM) statistics were determined by three experiments' data using one-way ANOVA with Tukey's post hoc test, ^∗∗∗^*p* < 0.001; ^∗∗^*p* < 0.01. n.s.: not significant, compared with the (RVSE untreated) samples.

Viral replication was investigated at the concentration of RVSE up to 100 *μ*g/mL, which did not show the cytotoxicity to MDCK cells (Figure 4(a)). Viral replication in MDCK cells treated with varying concentrations of RVSE and infected with A/PR/8/34-GFP was inhibited as measured by decreasing levels of GFP expression compared with those of untreated cells 24 h after infection (Figure 4(b)). Flow cytometry analysis using fluorescence detection showed that RVSE effectively inhibits viral replication (Figures 4(c) and 4(d)). Further, we investigated viral replication in RVSE-treated Caucasian human lung carcinoma A549 cells infected with A/PR/8/34-GFP (Figures 4(e) and 4(f)). RVSE-treated MDCK and A549 cells exhibited significantly reduced viral loads following infection with influenza virus.

**Figure 5:**
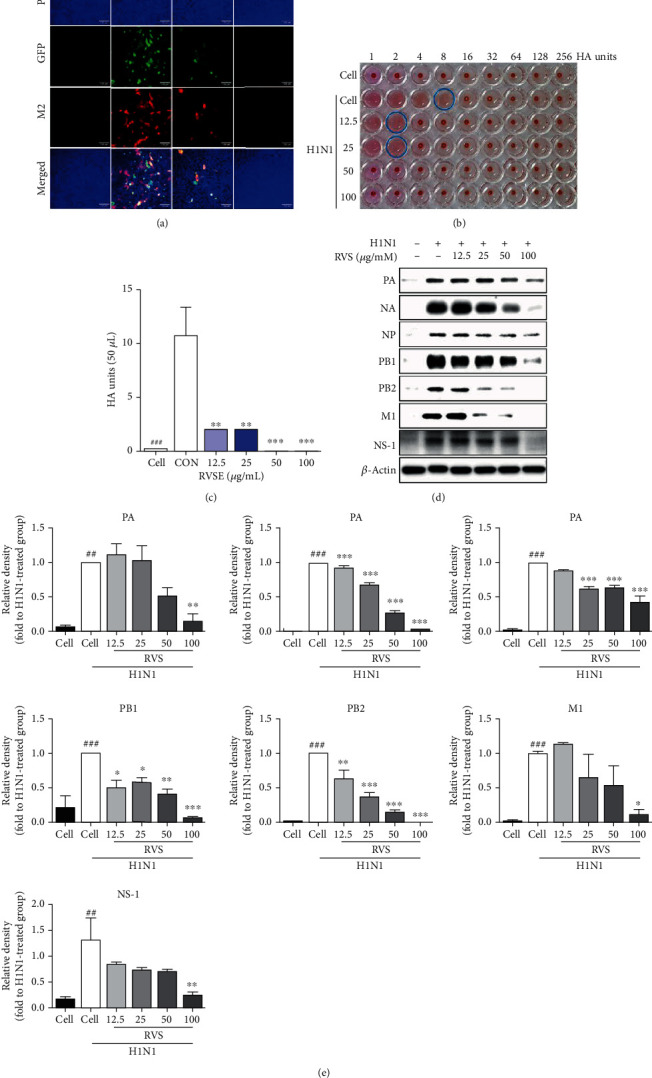
RVSE reduced the expression of influenza A virus proteins in infected MDCK cells. The reduction of M2 proteins in MDCK cells was observed with fluorescence microscopy using the influenza A virus protein M2-specific antibodies (a). MDCK cells were also stained with DAPI (blue), and the merged images represent M2 (red). Viruses were titrated from the supernatant via the hemagglutination inhibition assay. The supernatant titer of H1N1-infected cells treated with RVSE (12.5–100 *μ*g/mL) was significantly decreased compared with that without RVSE treatment (b, c). MDCK cells were cultured in 6-well plates (1 × 10^6^ cells/well) for 18 h. Then, H1N1 was mixed with different concentrations of RVSE (12.5, 25, 50, and 100 *μ*g/mL), and the mixtures were incubated at 37°C for 1 h. MDCK cells were infected with these mixtures at 37°C for 2 h. Afterwards, the virus was removed, the cells were washed three times with PBS, and the medium was replaced by complete DMEM. After 8 h, the cells were harvested, and western blotting was performed using the whole cell extracts. Influenza H1N1 virus protein levels (PA, NA, NP, PB1, PB2, M1, and NS-1) in MDCK cell lysates were detected using western blotting, and *β*-actin was analyzed as a loading control (d, e). The blots of NA and NS-1 were stripped and reprobed using *β*-actin antibody. The data are representative of three independent experiments that gave similar results. Bar graph (mean ± SEM) statistics were determined by three experiments' data using one-way ANOVA with Tukey's post hoc test, ^∗∗∗^*p* < 0.001; ^∗∗^*p* < 0.01. n.s.: not significant, compared with the (RVSE untreated) samples.

We also evaluated the effect of RVSE on expression of influenza A virus proteins, such as M2, using immunofluorescence analysis in RVSE-treated MDCK cells 24 h after infection with A/PR/8/34-GFP (Figure 5(a)). The expression of influenza A virus protein M2 was inhibited by RVSE concentrations of 25 and 100 *μ*g/mL following infection with A/PR/8/34-GFP at 24 h (Figure 5(a)).

We also confirmed that, compared with supernatant titers of H1N1-infected cells that were untreated (8 HAUs), titers of H1N1- and RVSE/H1N1-infected cells treated with 12.5 and 25 (2 HAUs) or 50 and 100 *μ*g/mL of RVSE (0 HAU) were significantly decreased (Figures 5(b) and 5(c)), indicating that RVSE inhibited A/PR/8/34-induced GFP expression and cell death in MDCK cells.

We monitored the regulation of influenza A virus protein expressions (PA, NA, NP, PB1, PB2, M1, and NS-1) by RVSE using western blot analysis at 24 h after infection with H1N1. The expression of influenza A virus proteins, PA, NA, NP, PB1, PB2, M1, and NS-1, was significantly inhibited in RVSE-treated MDCK cells (100 *μ*g/mL) upon infection with H1N1 at 24 h (Figures 5(d) and 5(e)).

### 3.5. Inhibitory Effect of the RVSE on the Influenza Virus *In Vivo*

**Figure 6:**
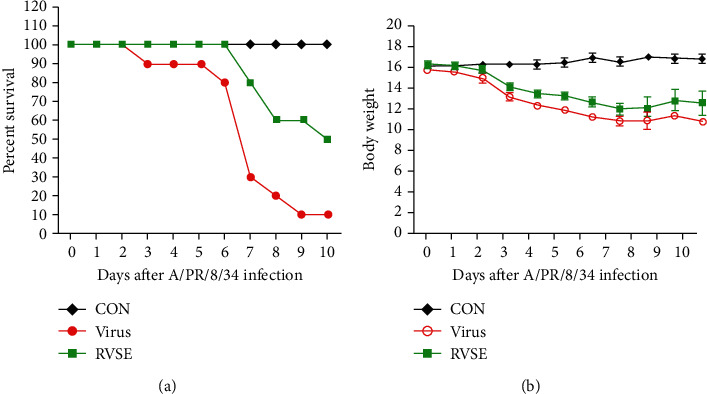
Effect of RVSE on influenza A virus infection in mice. BALB/c mice were treated orally with 10 mg/kg RVSE (200 *μ*L/mouse) 1, 2, 3, 4, 5, 6, 7, 8, 9, and 10 days after A/PR/8/34 virus infection. (a) Percent survival and (b) body weight were monitored daily until 10 days postinfection.

We initially examine the impact of RVSE on influenza A virus infection in mice. Mice, which were treated once daily with RVSE (10 mg/kg), maintained a relatively stable body weight, and no significant clinical symptoms were observed throughout the study (data not shown). Untreated A/PR/8/34-infected mice displayed significant body weight loss by 3 dpi before dying within 3 dpi (Figure 6). By contrast, RVSE-treated mice exhibited significantly increased survival after A/PR/8/34 infection (Figure 6(a)). Survival rate in the RVSE-treated group 10 dpi was 50%, higher than that in the viral control group (10%). Further, RVSE treatment did protect against body weight loss following viral infection (by approximately 11.5%) compared with the findings in untreated mice (Figure 6(b)).

### 3.6. Structural Elucidation of Ten Flavonoids

**Figure 7:**
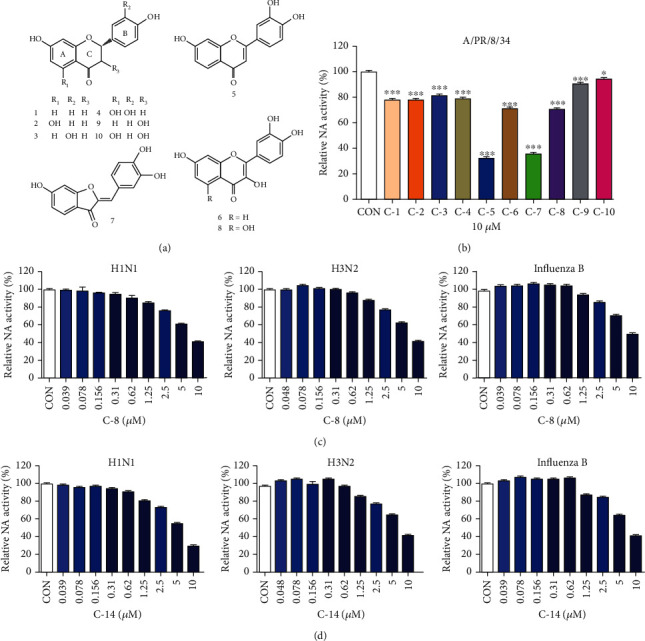
Determination of neuraminidase (NA) inhibition efficacy of the 10 components identified from RVSE. (a) Structures of 10 components of RVSE: 1, butin; 2, eriodictyol; 3, liquiritigenin; 4, naringenin; 5, 5-deoxyluteolin; 6, fisetin; 7, sulfuretin; 8, quercetin; 9, garbanzol; and 10, aromadendrin. (b) Measurement of the antiviral activity of RVSE components (10 *μ*M) using NA inhibition assay. The influenza A virus A/PR/8/34 was added to the indicated concentrations of RVSE components. Fluorescence was measured using fluorescence spectrophotometry (excitation, 365 nm; emission, 415–445 nm). The treatment with components (c) 5 and (d) 7 demonstrated the highest NA inhibitory activity against all viruses. Bar graph (mean ± SEM) statistics were determined by three experiments' data using one-way ANOVA with Tukey's post hoc test, ^∗∗∗^*p* < 0.001; ^∗^*p* < 0.05, compared with CON (untreated) preparations.

The structures of ten flavonoids, isolated from RVS, were elucidated by 1-D and 2-D NMR and mass spectrometry and compared with those of flavonoids reported in the literature (Figure 7(a)). Flavonoids were identified as butin (1) [33], eriodictyol (2) [34], liquiritigenin (3) [35], naringenin (4) [35], 5-deoxyluteolin (5) [36], fisetin (6) [37], sulfuretin (7) [38], quercetin (8) [33], garbanzol (9) [38], and aromadendrin (10) [35].

### 3.7. Anti-Influenza Efficacy of the Components Identified from RVSE

We further assessed NI efficacy of 10 components isolated from RVSE (Figures 7(a) and 7(b)). Components 5 and 7 (10 *μ*M) significantly decreased NA activity of A/PR/8/34 by 67.8% and 64.1%, respectively. These two components 5 and 7 inhibit NA activity effectively compared with other RVSE components. Inhibition of NA activity was confirmed using an NA-Fluor™ influenza NA assay. Components 5 and 7 (0–100 *μ*M) were mixed with viruses H1N1, H3N2, and influenza B. Oseltamivir carboxylate was used as a positive control. Treatment with components 5 and 7 induced the greatest inhibition of NA from all viruses (Figures 7(c) and 7(d)). Further, dose-dependent NI by components 5 and 7 is effective for NA for all influenza type A and B strains. These two constituents of RVSE are the major effective constituents for NI.

### 3.8. Quantification of Components 5 and 7 in RVSE by HPLC Analysis

**Figure 8:**
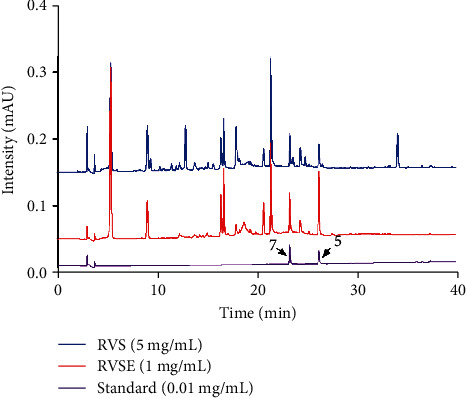
HPLC profiles of components in RVS (5 mg/mL) and RVSE (1 mg/mL) were monitored at 254 nm and compared with two standard compounds (5, 5-deoxyluteolin; 7, sulfuretin).

We used HPLC analysis of RVS and RVSE to quantify components 5 and 7 isolated from RVSE. HPLC profiles showed that components 5 and 7 were detected at 26.227 and 23.319 min, respectively (Figure 8). HPLC analysis also showed that RVS contained components 5 and 7 at 3.4 and 3.2 mg/g, and RVSE showed concentrations of 44 and 20 mg/g, respectively.

### 3.9. Protein–Ligand Docking Simulation and Pharmacophore Analysis of the Components in RVSE

**Figure 9:**
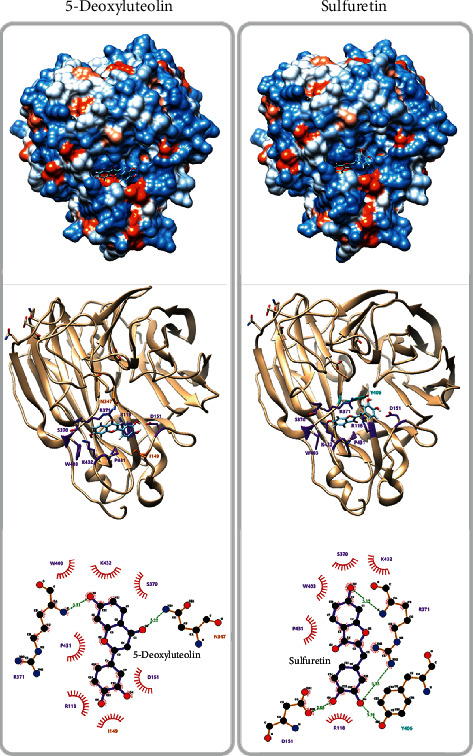
Protein docking simulation between NA and RVSE components. Binding affinity of components 5 and 7 with NA (09H1N1, PDB ID: 3TI6) was predicted by protein docking simulation using SwissDock. LigPlot+ software was applied to analyze their key hydrophobic and hydrogen bonds.

Oseltamivir carboxylate inhibits the release of replicated viruses from infected host cells by interacting with NA, and we thus investigated the molecular interactions between H1N1 NA (PDB code: 3TI6) and two components in RVSE with a protein–ligand docking simulation and pharmacophore analysis using SwissDock and LigPlot+ software. The pharmacophore analysis showed that 5-deoxyluteolin formed seven hydrophobic interactions and two hydrogen bonds and sulfuretin formed five hydrophobic bonds and four hydrogen bonds with NA (Figure 9). Specifically, 5-deoxyluteolin and sulfuretin were stably bound to NA by common molecular interactions: the hydrophobic interactions of AC-rings in 5 and 7 with S370, W403, and K432 and hydrogen bonds with R371; and hydrophobic interaction of B-ring in 5 and 7 with R118 and D151. 5-Deoxyluteolin and sulfuretin were further stabilized by I149, N347, and Y406. These two components were further stabilized by molecular interactions with I149 and N347 (5-deoxyluteolin) and Y406 (sulfuretin), respectively.

## 4. Discussion

An ongoing urgent medical need currently exists to develop new strategies to combat influenza virus infection [39–42]. Antiviral drugs are the only way to treat the disease when a vaccine is not available.

Currently, COVID-19 is causing deaths worldwide, and the number of cases of simultaneous COVID-19 and influenza virus infection is increasing. Such coinfection is common during periods of increased novel COVID-19 transmission [2–4]. Patients with simultaneous infection are at high risk of poor outcomes [2–4].

The NA is a glycoprotein present on the surface of the influenza virus. The enzyme is required for release of progeny virions from infected cells by cleaving sialic acid groups on the cell surface that bind to viral hemagglutinin. NA inhibitors prevent the release of progeny virions by interacting with the highly conserved active site of NA [14–16]. Unfortunately, H274Y and E119G/D/A mutations of NA decrease susceptibility to NA inhibitors oseltamivir and zanamivir, respectively, resulting in continued demand for the development of new agents [17, 18]. NA remains an attractive target for developing anti-influenza drugs.

New and effective preventive and therapeutic agents might be found among natural products [39–41]. We investigated the antiviral activity of plant extracts against influenza virus infection [19, 20]. We selected an ethanolic extract from the bark of RVS [43] for this study due to the beneficial effects of this plant on human health, which have already been reported in many literature. RVS has been used as a traditional herbal medicine for various symptoms, such as gastroenteritis, diabetes, arthritis, hypertension, stroke, and cancer [36, 43]. Previously, we demonstrated that RVS and its active constituents blocked immune checkpoint PD-1/PD-L1 CTLA-4/CD80 [21]. The antiviral efficacy of RVS has been investigated, but not for influenza virus [22, 23].

We examined the antiviral activity of RVS against influenza virus and found that treatment with RVS markedly reduced viral replication in MDCK cells, evaluated using a GFP-tagged virus. Also, RVS displayed significant inhibitory activity against NA from A/PR/8/34. Subsequently, we isolated an RVS fraction with substantial antiviral activity via NI. Three fractions were prepared from the ethanolic crude extract of RVS. *In vitro* assays indicate that RVSE is more potent than H_2_O and CHCl_3_ fractions. We confirm that RVSE significantly suppresses influenza virus infection in MDCK and A549 cells through concentration-dependent NI. RVSE treatment also inhibited expression of virus proteins, PA, NA, NP, PB1, PB2, M1, and NS-1, and decreased mortality in mice exposed to the influenza A/PR/8/34 virus by 50% and prevented weight loss by approximately 11.5% (Figures 6(a) and 6(b)).

Active compounds in RVSE were isolated, and 10 components were identified. 5-Deoxyluteolin (5) and sulfuretin (7) have the highest inhibitory activity against NA. The strong inhibition of NA activity of RVSE likely results from high concentrations of components 5 and 7.

**Figure 10:**
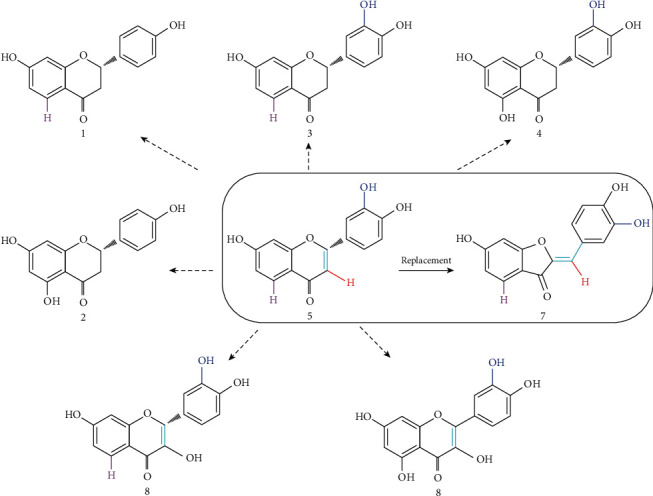
Identification of structure–activity relationship for NA inhibitory activity in compounds from RVSE.

Structure–activity relationships among isolated flavonoids indicate that components 5–8 with a double bond between C-2 and C-3 show greater inhibition of NA activity than components 1–4. This finding suggests the double bond at C-2/3 is a key functional element. Components 5 and 7 are not substituted at C-3, compared with components 6 and 8 that display a hydroxyl group at C-3. Thus, lack of substitution at C-3 might be an important functional feature (Figure 10). These findings may be useful in evaluating the structure–activity relationships of other flavonoids for anti-influenza activity.

We identified constituents of RVSE using HPLC and confirmed that components 5 and 7 inhibit NA activity using an NA-Fluor™ influenza NA assay. We also examined amounts of identified phytochemicals and found that active components 5 and 7 were relatively abundant in RVSE, at 44 and 20 mg/g, respectively. We show some evidence that the antiviral effects of RVSE and its components are due to these components. NA sequences among viral strains may provide a more interesting interpretation for anti-influenza activity of components 5 and 7.

In the early 2000s, NA inhibitor-resistant influenza viruses emerged by the mutations in E119, H274, R292, and N294 of NA [44, 45]; thus, the research that investigates antiviral efficacy of natural products, including RVSE and components 5 and 7, against these resistant viruses will be of interest.

In summary, we demonstrate that RVSE significantly averted influenza virus infection in MDCK and A549 cells by NI. Further, RVSE treatment decreased mortality in mice exposed to influenza A/PR/8/34 virus and prevents weight loss compared with that in untreated mice. Further, we confirmed that 5-deoxyluteolin and sulfuretin (components 5 and 7) inhibited NA activity notably among the ten components isolated and identified from RVSE. RVS, RVSE, and its components are effective in inhibiting the NA activity of both influenza virus A and B. RVSE and its components may provide good candidates and building blocks for novel anti-influenza drugs. However, additional mechanistic and *in vivo* studies are required to elucidate in detail the mode of action of active components 5 and 7.

## Data Availability

The datasets generated and/or analyzed during the present study are available from the corresponding author on reasonable request.

## Supplementary Materials

Supplementary Materials Figure S1: ^1^H and ^13^C NMR spectrum of compound 1 in methanol-*d*_4_ (600 and 150 MHz). Figure S2: ^1^H and ^13^C NMR spectrum of compound 2 in methanol-*d*_4_ (600 and 150 MHz). Figure S3: ^1^H and ^13^C NMR spectrum of compound 3 in methanol-*d*_4_ (600 and 150 MHz). Figure S4: ^1^H and ^13^C NMR spectrum of compound 4 in methanol-*d*_4_ (600 and 150 MHz). Figure S5: ^1^H and ^13^C NMR spectrum of compound 5 in methanol-*d*_4_ (600 and 150 MHz). Figure S6: ^1^H and ^13^C NMR spectrum of compound 6 in methanol-*d*_4_ (600 and 150 MHz). Figure S7: ^1^H and ^13^C NMR spectrum of compound 7 in methanol-*d*_4_ (600 and 150 MHz). Figure S8: ^1^H and ^13^C NMR spectrum of compound 8 in methanol-*d*_4_ (600 and 150 MHz). Figure S9: ^1^H and ^13^C NMR spectrum of compound 9 in methanol-*d*_4_ (600 and 150 MHz). Figure S10: ^1^H and ^13^C NMR spectrum of compound 10 in methanol-*d*_4_ (600 and 150 MHz).
