# Supplementary material for: Anti-Influenza Activity of an Ethyl Acetate Fraction of a Rhus verniciflua Ethanol Extract by Neuraminidase Inhibition
Source: Oxid Med Cell Longev. 2020 Oct 29;2020:8824934. doi: 10.1155/2020/8824934 (PMC7661131; doi:10.1155/2020/8824934)
Supplement: Supplementary Materials — Figure S1: 1H and 13C NMR spectrum of compound 1 in methanol-d4 (600 and 150 MHz). Figure S2: 1H and 13C NMR spectrum of compound 2 in methanol-d4 (600 and 150 MHz). Figure S3: 1H and 13C NMR spectrum of compound 3 in methanol-d4 (600 and 150 MHz). Figure S4: 1H and 13C NMR spectrum of compound 4 in methanol-d4 (600 and 150 MHz). Figure S5: 1H and 13C NMR spectrum of compound 5 in methanol-d4 (600 and 150 MHz). Figure S6: 1H and 13C NMR spectrum of compound 6 in methanol-d4 (600 and 150 MHz). Figure S7: 1H and 13C NMR spectrum of compound 7 in methanol-d4 (600 and 150 MHz). Figure S8: 1H and 13C NMR spectrum of compound 8 in methanol-d4 (600 and 150 MHz). Figure S9: 1H and 13C NMR spectrum of compound 9 in methanol-d4 (600 and 150 MHz). Figure S10: 1H and 13C NMR spectrum of compound 10 in methanol-d4 (600 and 150 MHz). [file 8824934.f1.docx]

**Supporting Information**

**Anti-influenza activity of an ethyl acetate fraction of a *Rhus verniciflua* ethanol extract by neuraminidase inhibition**

**Young Soo Kim^†^, Wei Li^†^, Ji Hye Kim, Hwan-Suck Chung, Jang-Gi Choi^*^**

Korea Institute of Oriental Medicine (KIOM), Korean Medicine (KM) Application Center, 70 Cheomdan-ro, Dong-gu, Daegu, 41062, Republic of Korea

^*^ Corresponding Author: E-mail: [Jang-gichoi@kiom.re.kr](mailto:Jang-gichoi@kiom.re.kr)

^†^ Contributed equally


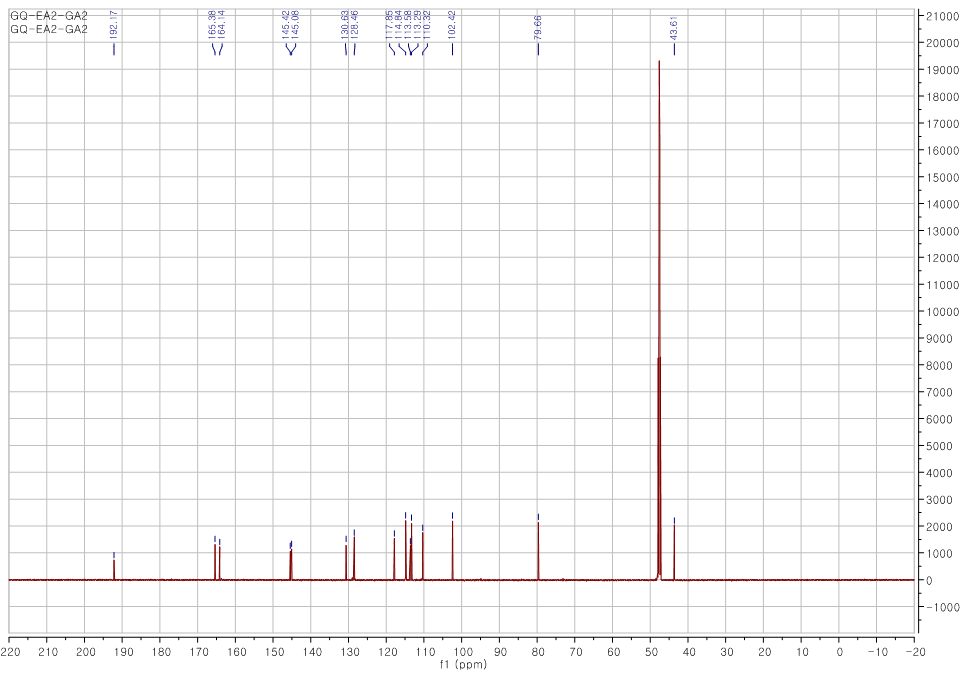

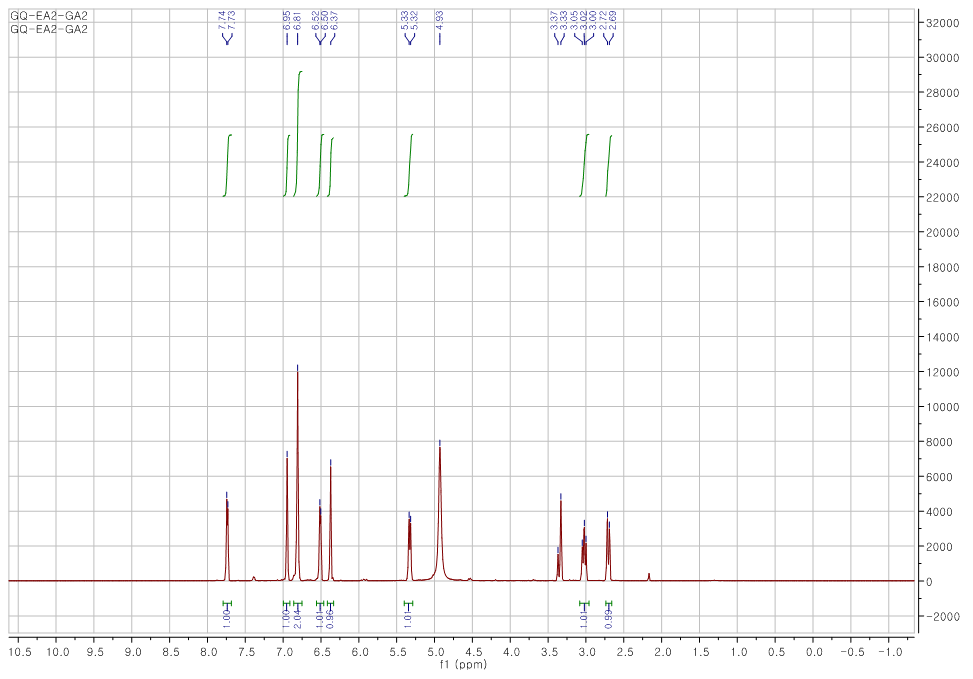


**Figure S1.** ^1^H and ^13^C NMR spectrum of compound **1** in methanol-*d_4_* (600 and 150 MHz)


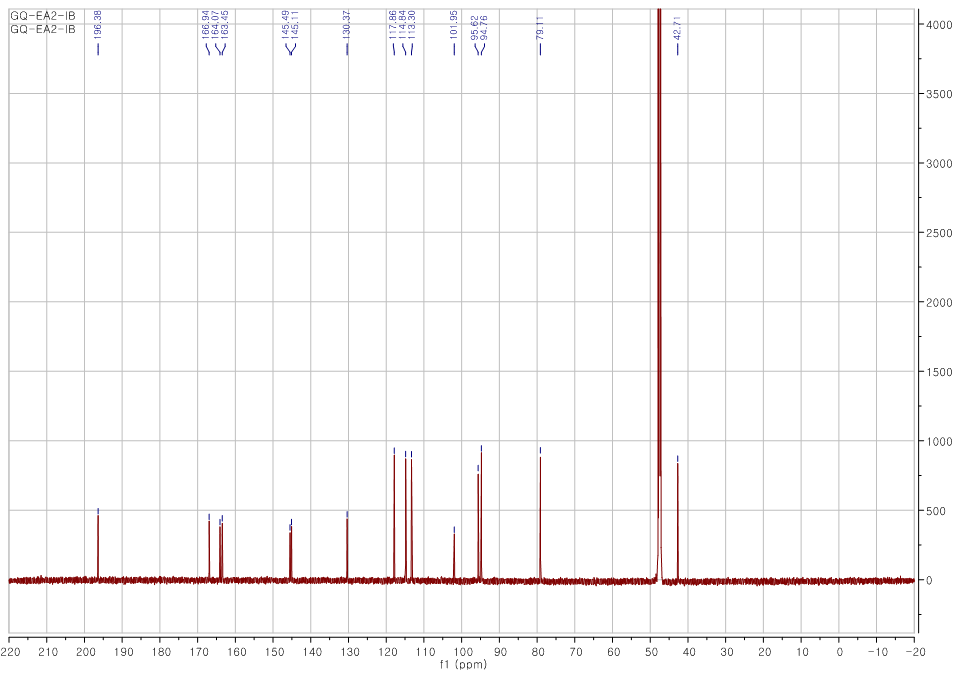

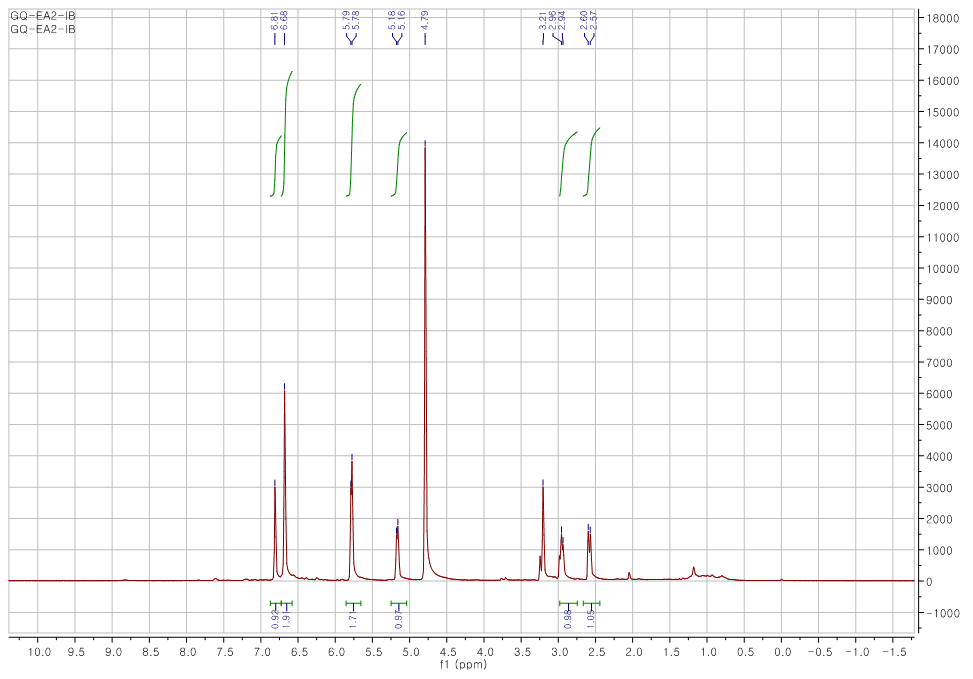


**Figure S2.** ^1^H and ^13^C NMR spectrum of compound **2** in methanol-*d_4_* (600 and 150 MHz)


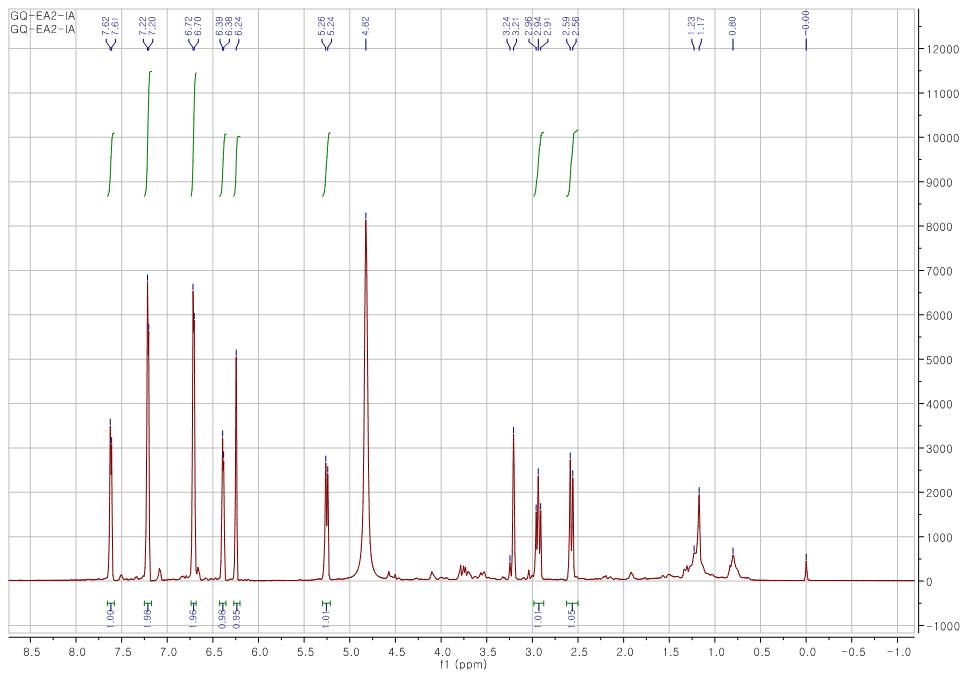

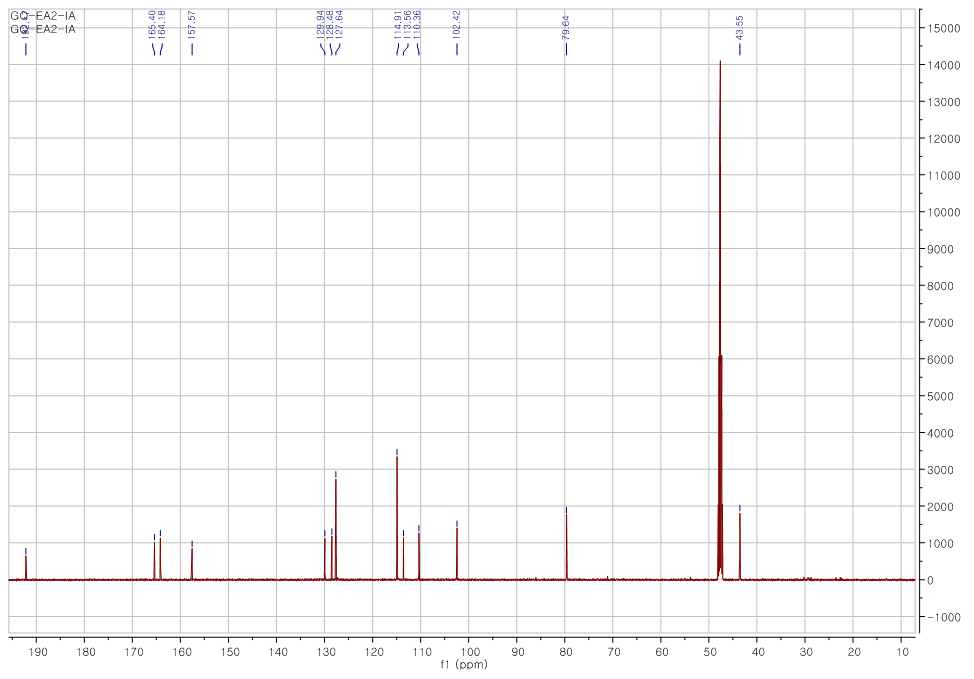


**Figure S3.** ^1^H and ^13^C NMR spectrum of compound **3** in methanol-*d_4_* (600 and 150 MHz)


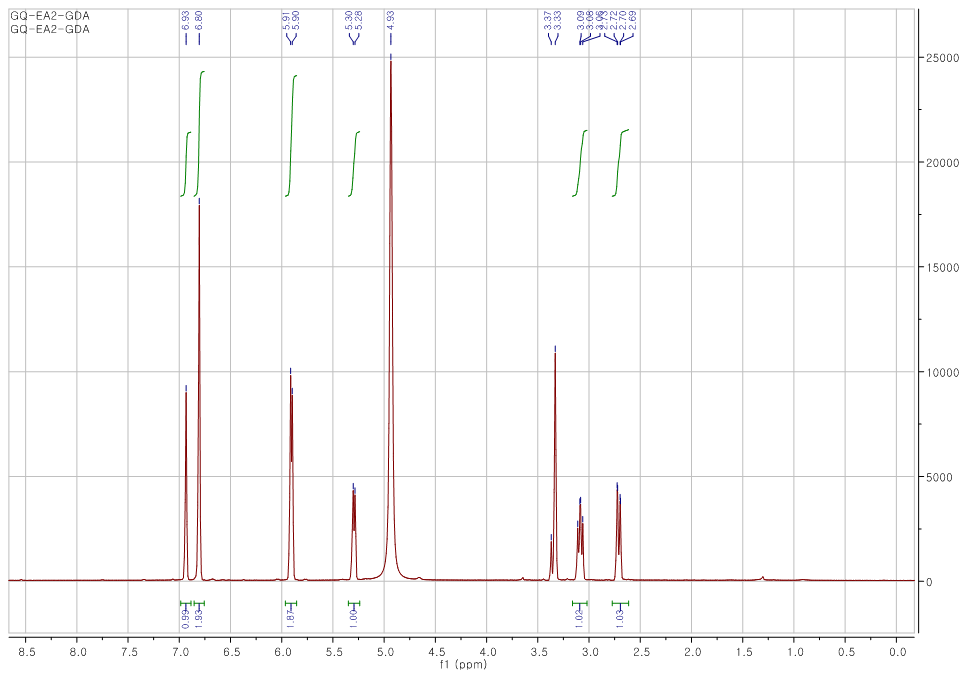

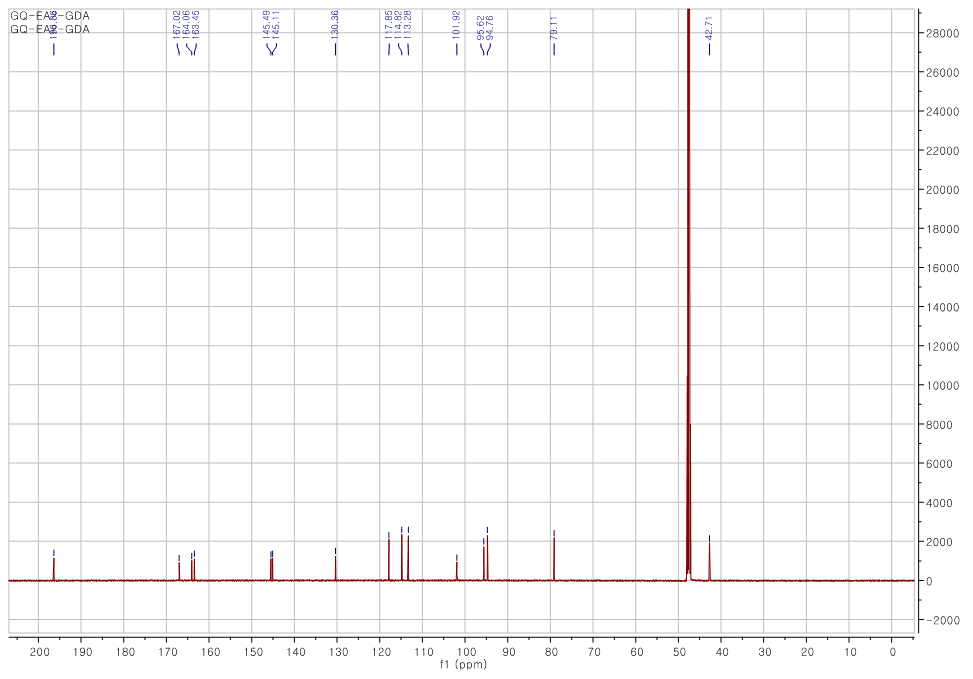


**Figure S4.** ^1^H and ^13^C NMR spectrum of compound **4** in methanol-*d_4_* (600 and 150 MHz)


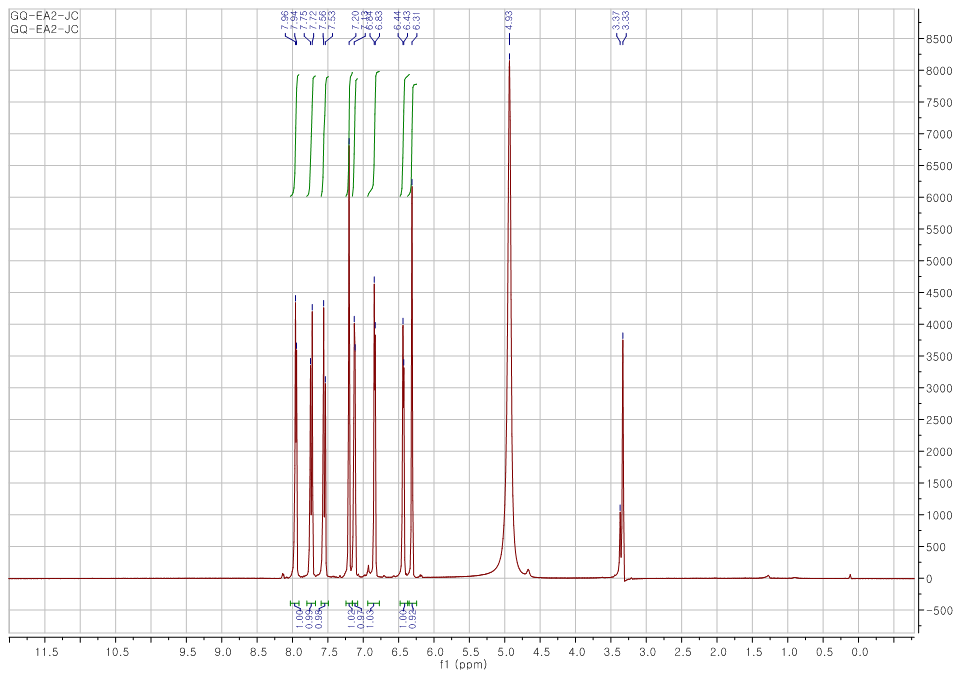

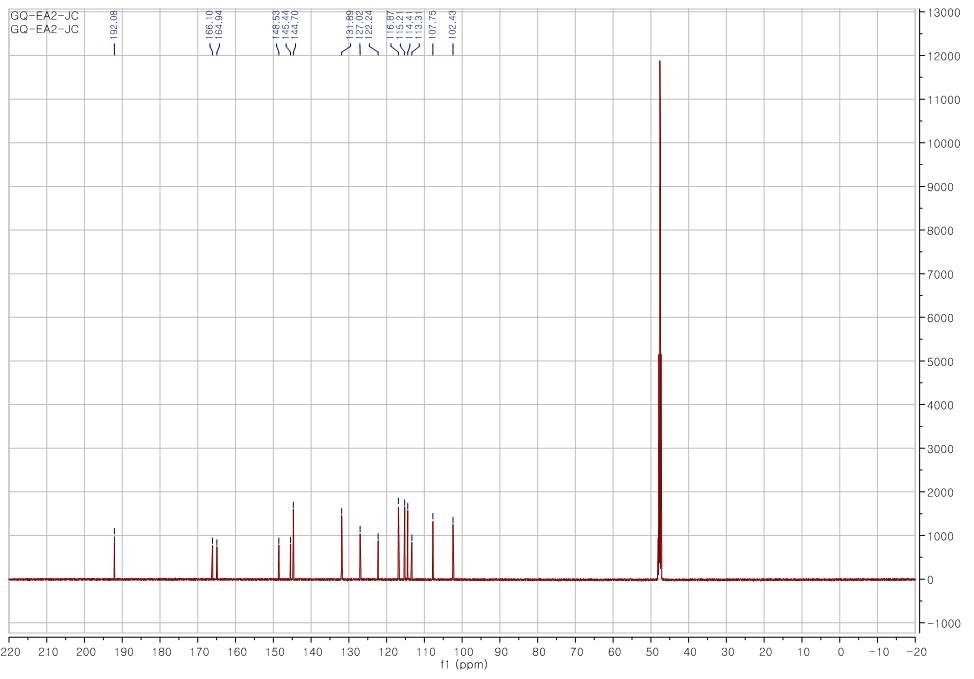


**Figure S5.** ^1^H and ^13^C NMR spectrum of compound **5** in methanol-*d_4_* (600 and 150 MHz)


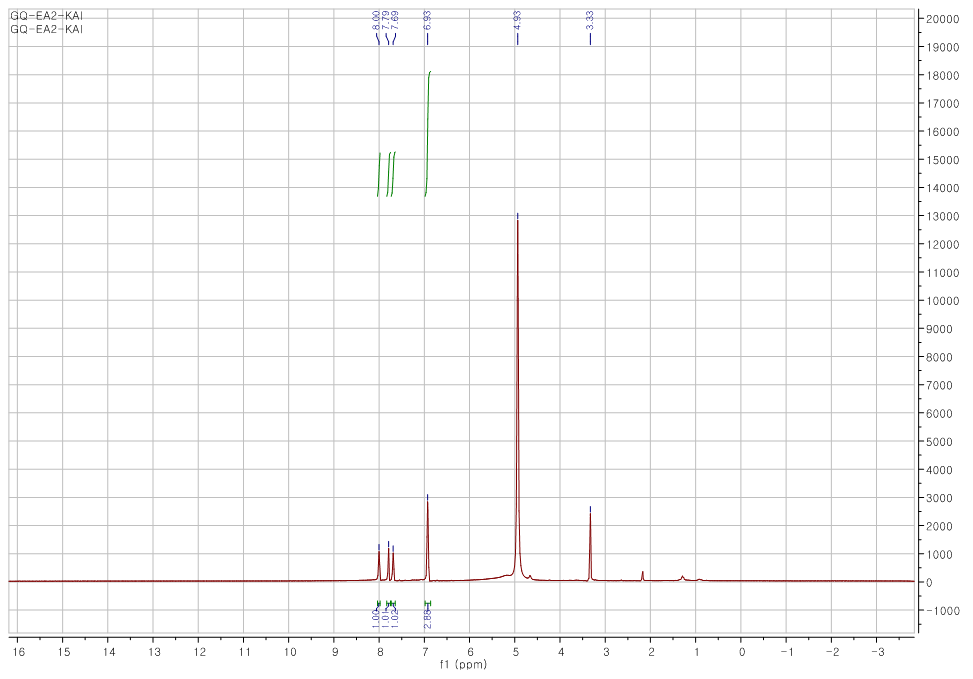

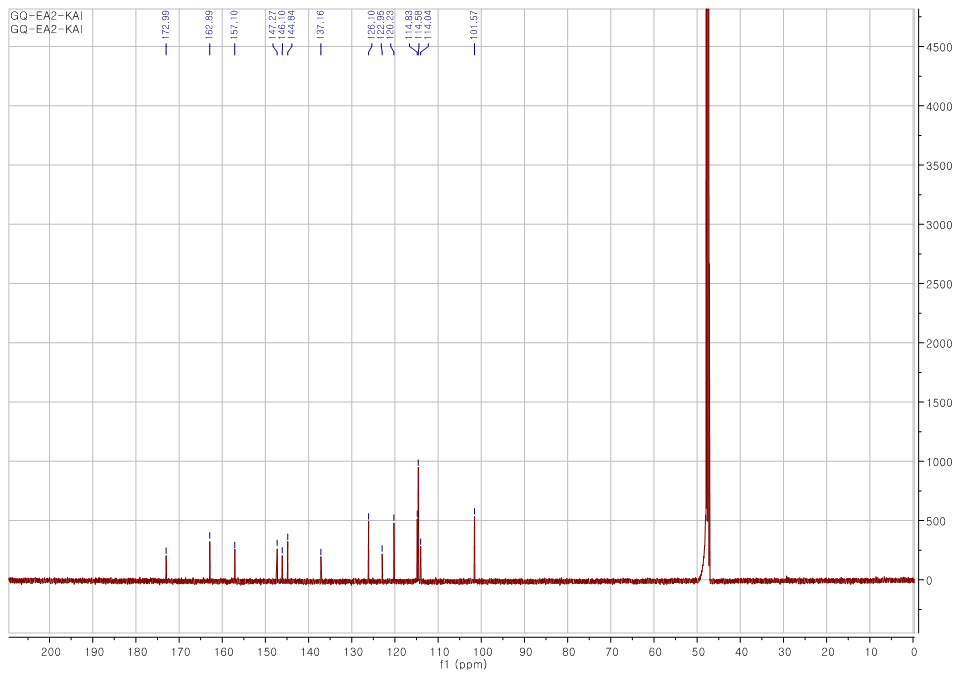


**Figure S6.** ^1^H and ^13^C NMR spectrum of compound **6** in methanol-*d_4_* (600 and 150 MHz)


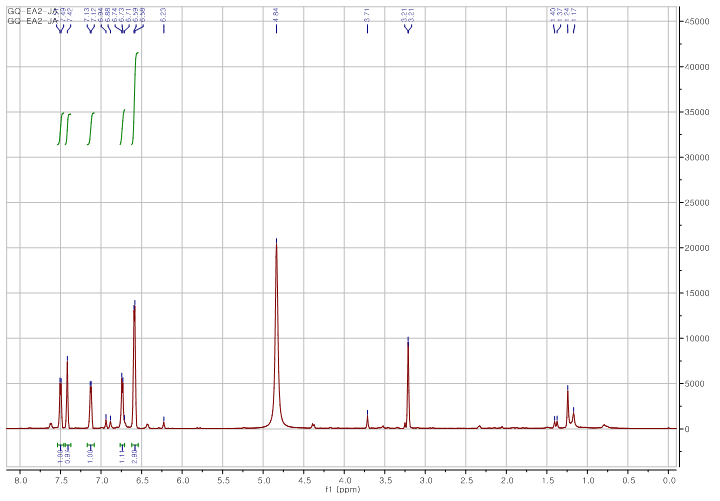

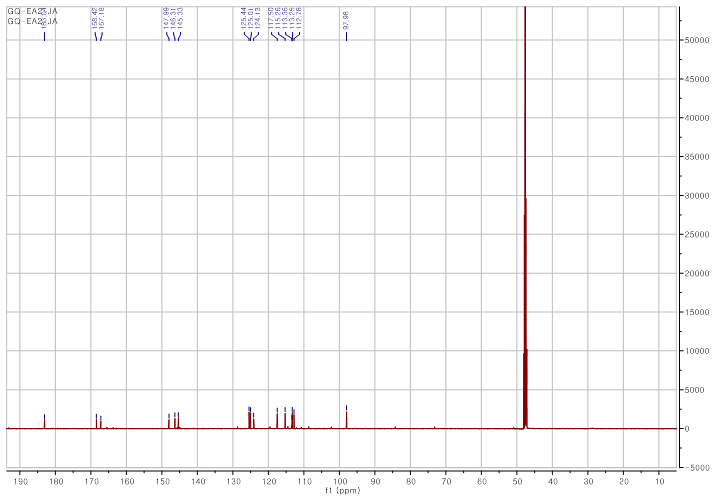


**Figure S7.** ^1^H and ^13^C NMR spectrum of compound **7** in methanol-*d_4_* (600 and 150 MHz)


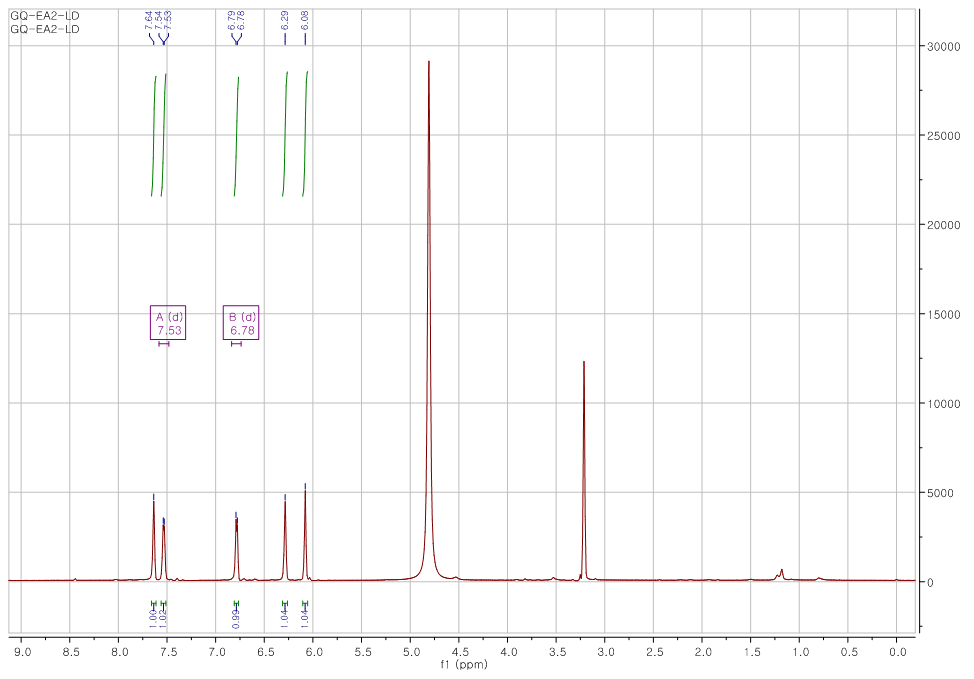

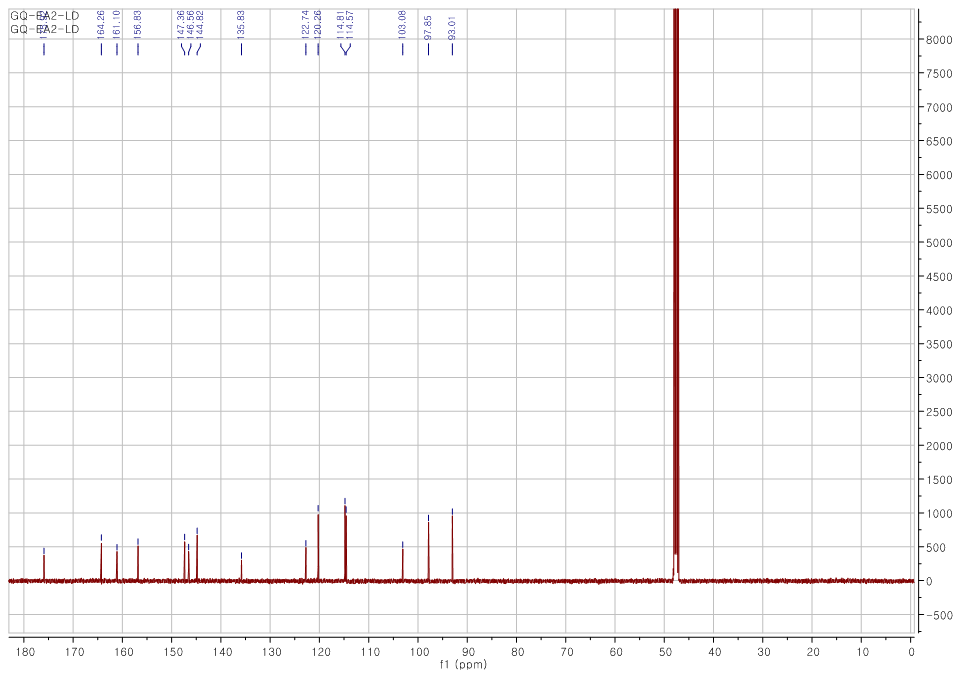


**Figure S8.** ^1^H and ^13^C NMR spectrum of compound **8** in methanol-*d_4_* (600 and 150 MHz)


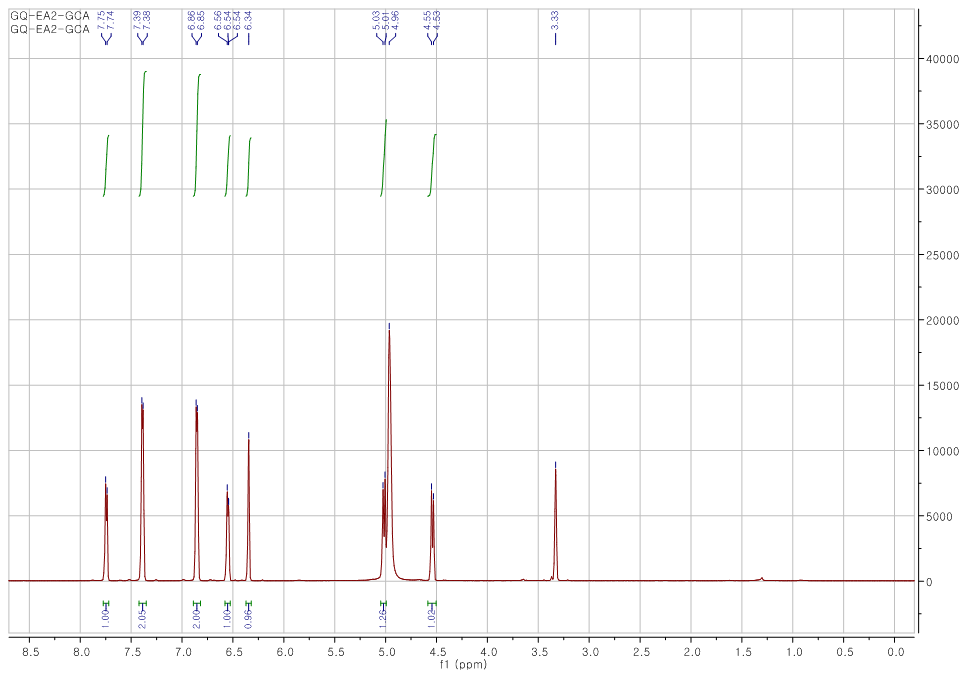

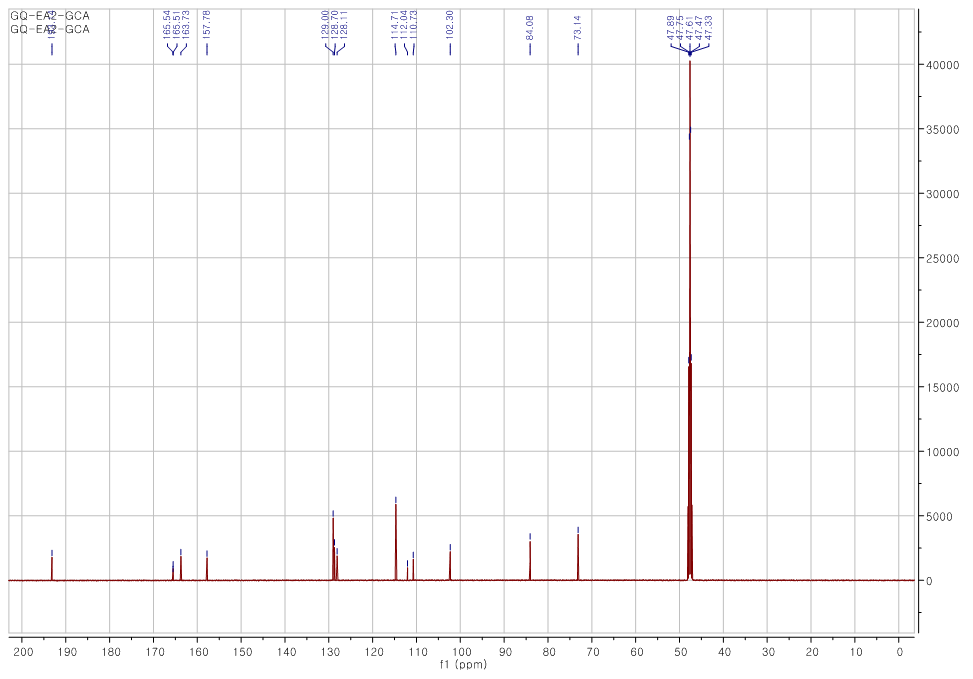


**Figure S9.** ^1^H and ^13^C NMR spectrum of compound **9** in methanol-*d_4_* (600 and 150 MHz)


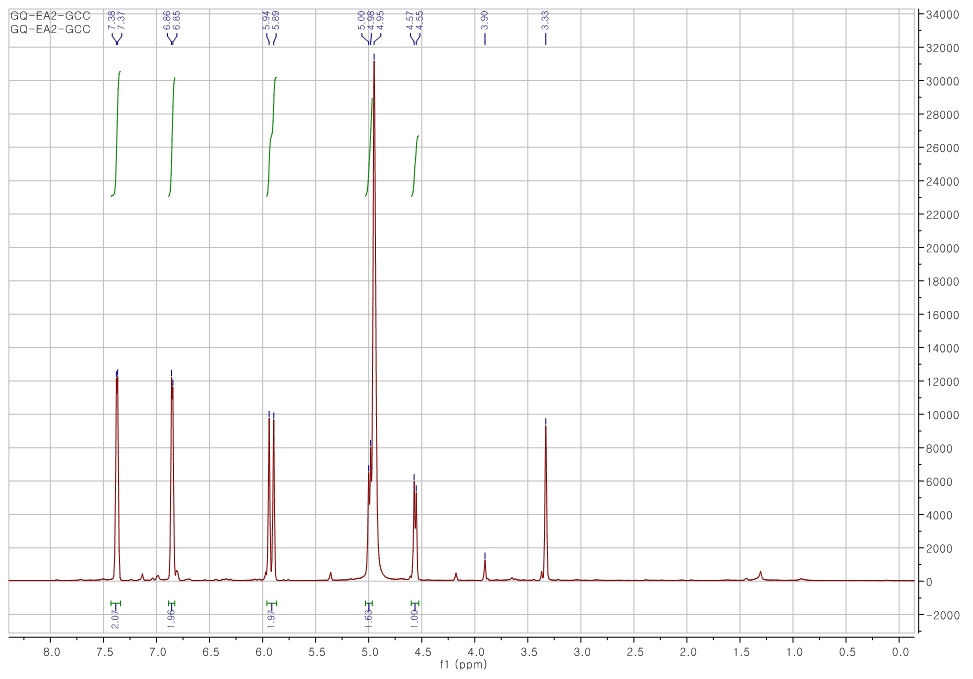

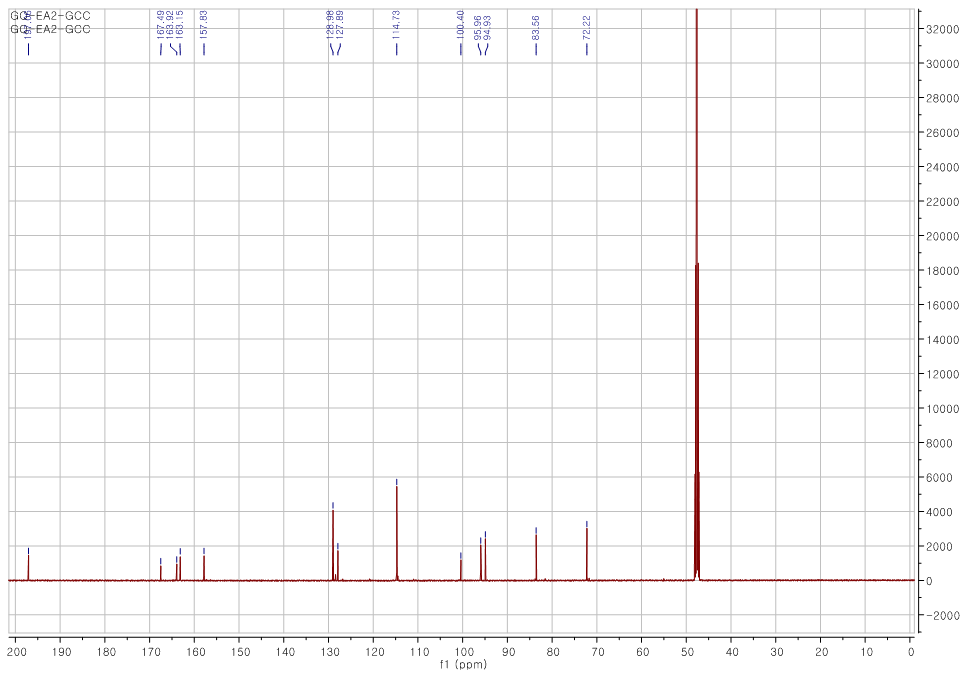


**Figure S10.** ^1^H and ^13^C NMR spectrum of compound **10** in methanol-*d_4_* (600 and 150 MHz)
